# Neutrophil-Lymphocyte Ratio as a Potential Biomarker for Schizophrenia Severity: A Cross-Sectional Study

**DOI:** 10.7759/cureus.93204

**Published:** 2025-09-25

**Authors:** Rashmi Meena, Ragini Meena, Jiwan S Meena

**Affiliations:** 1 Pathology, Hamidia Hospital/Gandhi Medical College, Bhopal, IND; 2 Obstetrics and Gynaecology, Jaya Arogya Hospital/Gajra Raja Medical College, Gwalior, IND; 3 Community Medicine, Gandhi Medical College, Bhopal, IND

**Keywords:** antipsychotic treatment, biomarker, bprs score, complete blood count, inflammation, medical comorbidities, neutrophil-lymphocyte ratio, schizophrenia, symptom severity

## Abstract

Background: Schizophrenia is a chronic psychiatric disorder with a complex etiology, and inflammation has been increasingly implicated in its pathophysiology. The neutrophil-lymphocyte ratio (NLR), a simple hematological marker, may serve as an adjunctive biomarker for disease severity.

Objective: To evaluate the association between NLR and schizophrenia severity, and to compare inflammatory status between treated and untreated patients.

Methods: A hospital-based cross-sectional study was conducted among 100 patients with International Classification of Diseases, Tenth Revision (ICD-10)-diagnosed schizophrenia. Of 143 patients screened, 43 were excluded. Symptom severity was assessed using the Brief Psychiatric Rating Scale (BPRS). Complete blood counts were analyzed to calculate NLR. Statistical analysis included Welch’s t-test, chi-square test, and Pearson’s correlation (α = 0.05).

Results: The mean age of participants was 47.9 ± 8.6 years; 73% were male. Fifty-nine patients were treated, and 41 were untreated. Untreated patients had significantly higher NLR values (8.94 ± 5.08 vs. 4.57 ± 1.90; p < 0.001). The mean BPRS score was 61.9 ± 17.3. NLR correlated strongly with BPRS (r = 0.736, p < 0.001).

Conclusion: Elevated NLR is associated with greater schizophrenia severity and higher values in untreated patients. NLR, being inexpensive and widely available, could serve as an adjunctive tool for routine monitoring and personalized management.

## Introduction

Schizophrenia is a chronic and serious mental disorder that affects how a person thinks, feels, and behaves. It is characterized by a range of symptoms, including delusions, hallucinations, disorganized thinking, and cognitive impairment [1]. Its etiology involves genetic predisposition, environmental triggers, neurotransmitter dysregulation, and immune dysfunction [2]. The disorder usually begins in late adolescence or early adulthood, with men tending to exhibit symptoms earlier than women [3]. The exact pathophysiology remains unclear, but evidence points to neuroinflammation and immune dysregulation as key factors [4]. The inflammatory hypothesis of schizophrenia proposes that systemic inflammation contributes to neurodevelopmental abnormalities, neurotransmitter imbalances, and neuronal damage, ultimately leading to the onset and exacerbation of symptoms [4]. The link between inflammation and psychiatric disorders has been extensively studied. Conditions such as major depressive disorder, bipolar disorder, and schizophrenia have all been associated with elevated inflammatory markers [4-6]. Recent evidence supports the inflammatory hypothesis of schizophrenia, linking systemic immune activation with neurodevelopmental abnormalities and symptom severity [7]. Haematological markers, including the neutrophil-to-lymphocyte ratio (NLR), provide a simple and practical approach to assessing systemic inflammation. The NLR is determined by dividing the absolute neutrophil count by the absolute lymphocyte count, where elevated values reflect an increased inflammatory response [8]. An increase in NLR suggests a heightened inflammatory response, while a lower NLR may indicate a weakened immune function. Given its accessibility, NLR has been extensively investigated as a biomarker in multiple disease conditions [8]. In healthy individuals, the NLR generally ranges between 1.5 and 3.5, though variations occur with age, gender, ethnicity and physiological states such as pregnancy or stress [8]. Increasing evidence links systemic inflammation to psychiatric disorders, particularly schizophrenia, where elevated NLR supports the neuroinflammation hypothesis [9,10]. Higher NLR levels have been associated with greater symptom severity and cognitive deficits, while reductions have been reported following antipsychotic treatment [4,10]. These findings position NLR as a potential biomarker for disease monitoring, treatment response, and prognosis in schizophrenia [8,9,11]. 

Evidence of elevated NLR has been found in schizophrenia. Studies have found higher NLR in schizophrenia patients compared to healthy individuals, suggesting an ongoing inflammatory process. Increased NLR levels are positively correlated with symptom severity, particularly negative symptoms and cognitive impairments. Higher NLR is associated with treatment resistance and more severe psychotic episodes [12-14]. The mechanisms connecting NLR and schizophrenia may include neuroinflammation from increased neutrophil counts, lymphopenia indicating immune suppression, and blood-brain barrier dysfunction that permits peripheral cytokines to influence brain function [15,16]. Emerging psychiatric literature suggests elevated NLR in schizophrenia patients compared to healthy controls and possible associations with psychosis severity [17,18]. Persistent inflammation has also been implicated in negative symptoms and cognitive deficits, likely through its impact on synaptic plasticity and neurotransmitter function [19,20].

The Brief Psychiatric Rating Scale (BPRS) is one of the most widely used instruments for assessing symptom severity in schizophrenia [21]. It allows clinicians to evaluate both positive and negative symptom domains and to track changes over time [22,23]. Clinically, the BPRS assists in initial diagnosis, guides treatment planning, monitors therapeutic response, and facilitates the early detection of relapse [22,24]. Higher scores denote greater symptom severity, making the scale a valuable tool for both research and clinical practice. Given the established association between inflammation and schizophrenia severity, coupled with the accessibility of NLR as a biomarker, this study was undertaken to investigate the relationship between NLR and clinical severity as measured by the BPRS, and to compare these parameters between medicated and unmedicated patients with schizophrenia.

## Materials and methods

The present study was designed as a cross-sectional, hospital-based observational study, carried out jointly in the Department of Pathology and the Department of Psychiatry at Gandhi Medical College and the associated Hamidia Hospital in Bhopal, Madhya Pradesh, India. The study population consisted of patients attending both the outpatient and inpatient departments. Recruitment was conducted over an 18-month period from May 2023 to October 2024. Eligible participants were adults aged between 18 and 70 years who had a confirmed diagnosis of schizophrenia according to International Classification of Diseases, Tenth Revision (ICD-10) criteria [25] and were considered clinically stable enough to participate in the study. The study included both patients receiving antipsychotic treatment and those not currently on treatment. For the untreated group, eligibility additionally required that they had not taken any antipsychotic or sedative-hypnotic medication within the six months preceding enrollment. Written informed consent was obtained from all participants, and in cases where the patient was unable to provide consent, it was obtained from their legal guardians.

Patients with severe brain diseases, autoimmune conditions, cardiovascular disorders, active or chronic infections, or hematological abnormalities unrelated to schizophrenia were excluded. Additional exclusion criteria included recent use of anti-inflammatory drugs, corticosteroids, antibiotics, immunosuppressants, or lithium carbonate within the preceding four weeks, as well as pregnancy or lactation. A total of 143 patients were screened for eligibility, of whom 43 did not meet the inclusion criteria. The final study population therefore comprised 100 patients with schizophrenia who fulfilled the ICD-10 diagnostic criteria. Based on medication history, participants were categorized into two groups: medicated patients, who were currently receiving antipsychotic treatment, and unmedicated patients, who had not received any antipsychotic therapy for at least six months prior to enrollment.

Sample size estimation was initially attempted using the single-proportion formula for the reported prevalence of schizophrenia in India as 0.3% (p = 0.003) [26], which yielded a minimum sample size of 18. However, this approach was not appropriate for a hospital-based cross-sectional study with continuous outcomes. Therefore, an a priori power analysis was performed using Welch’s t-test for independent means, with α = 0.05 and 90% power. Based on meta-analytic data reporting standardized mean differences of 0.65-0.75 for NLR in schizophrenia [27,28], we assumed a conservative effect size of Cohen’s d = 0.7, consistent with prior meta-analytic evidence showing moderate-to-large standardized mean differences in NLR among schizophrenia patients compared to controls [27,28]. According to Cohen’s conventional benchmarks, d = 0.7 represents a large effect size [29].

For the standard deviation estimate, prior studies have reported variable values: Sugita et al. (2024) [30] observed a higher SD of 2.9 among acutely ill inpatients, while Leung et al. (2023) [31] reported a narrower SD of 1.9 in a large first-episode psychosis cohort. To adopt a conservative approach that accommodates both inpatient and outpatient variability, we selected an intermediate SD of 2.5 for our calculation. Using these parameters, the required sample size was 43 per group (86 total). To account for potential group imbalance, dropouts, and secondary correlation analyses, the final target was increased to 100, which provided >90% power to detect group differences as well as correlations of r ≥ 0.3 between NLR and clinical scales.

Data were collected using a structured, close-ended proforma that included socio-demographic details, clinical history, and laboratory parameters. Information was obtained through direct patient interviews, psychiatric evaluation reports, and hospital records. Symptom severity was assessed by a trained psychiatrist using the 18-item BPRS, with each item scored on a 7-point Likert scale ranging from 1 (not assessed) to 7 (extremely severe), yielding a total score range of 18 to 126, where higher scores indicate greater symptom severity [7,22,23]. Although the Positive and Negative Syndrome Scale (PANSS) is recognized for its extensive coverage of schizophrenia symptoms, we opted to use BPRS due to its brevity and logistical feasibility in our clinical setting. PANSS is a structured interview that requires approximately 45-50 minutes to administer, and interviewers must be trained to achieve standardized reliability [32,33]. In contrast, the BPRS can typically be completed in about 20 minutes, making it substantially shorter and more practical for routine clinical use while retaining robust psychometric reliability [7,34].

For laboratory investigations, a 3 mL venous blood sample was collected from the antecubital vein under aseptic precautions, placed in EDTA tubes, and analyzed in the Department of Pathology using an automated hematology analyzer. From the complete blood count (CBC) results, inflammatory markers including the NLR were calculated.

Statistical analysis involved descriptive measures such as means, standard deviations, and percentages. Between-group comparisons of continuous variables were performed using Welch’s t-test, while categorical variables were analyzed with the Chi-square test where appropriate. Correlation analysis was conducted using Pearson’s correlation coefficient. A p-value of less than 0.05 was considered statistically significant. All statistical analyses were performed using Epi Info version 7.2.7 (CDC, Atlanta, GA, USA), while Microsoft Office Excel (Microsoft, Redmond, WA, USA) was used for data entry, management, and descriptive statistics.

## Results

Table 1 shows the sociodemographic and clinical characteristics of the study participants (n = 100). A total of 100 patients diagnosed with schizophrenia were included in the analysis. The majority were male (73%), aged between 41-50 years (37%), and residing in urban areas (52%). Educational status varied, with 54% having primary or high school education, and 8% being illiterate. The socio-economic classification of the patients was assessed using the Modified Kuppuswamy scale, which categorizes individuals based on education, occupation, and family income [35]. Socioeconomic status was most frequently reported as lower middle (33%) or upper middle (27%). Forty-two percent of the sample reported current smoking, while 31% had at least one comorbidity. Regarding illness duration, 18% were newly diagnosed, while 64% had been living with schizophrenia for one to six years.

**Table 1:** Sociodemographic (Part A) and clinical (Part B) characteristics of the overall study population (n = 100). n = number of patient, SES = socioeconomic status

Variable	Category		n	%
Part A: Socio-demographic Profile				
Age	31–40 yrs		23	23
	41–50 yrs		37	37
	51–60 yrs		34	34
	61–70 yrs		6	6
Gender	Male		73	73
	Female		27	27
Residence	Rural		48	48
	Urban		52	52
Education		Primary/High School	54	54
		Senior Secondary	25	25
		Graduate / Postgraduate	13	13
		Illiterate	8	8
SES (Kuppuswamy)	Lower Middle		33	33
	Upper Middle		27	27
	Upper Lower		25	25
	Lower		15	15
Smoking	Yes		42	42
	No		58	58
Comorbidities	Present (Any)		31	31
	None		69	69
Duration of illness	Newly diagnosed		18	18
	1-3 years		23	23
	4-6 years		23	23
	7-9 years		18	18

Table 2 presents the distribution of patients by medication status. A majority (59%) were on antipsychotic medication, while 41% were unmedicated, indicating a substantial proportion of untreated or non-adherent cases.

**Table 2:** Medication status of study participants (n = 100) n = number of patients.

Medication Status	n	(%)
Medicated	59	(59)
Unmedicated	41	(41)
Total	100	(100)

Table 3 shows the associations between sociodemographic/clinical variables and medication use. Residence was significantly associated with treatment status (χ² = 3.85, p = 0.0498), with urban patients more likely to be medicated. Education also showed a significant association (χ² = 12.05, p = 0.017), with patients having senior secondary education demonstrating the highest treatment uptake. Duration of illness was strongly related to treatment status (χ² = 32.31, p < 0.001), as all newly diagnosed patients were untreated. No significant association was observed between socioeconomic status and treatment use (χ² = 0.045, p = 0.997).

**Table 3:** Association between socio-demographic/clinical factors and medication use SES = socioeconomic status

Variable	Chi-square	df	p-value	Interpretation
Residence	3.85	1	0.0498	Medication status was associated with urban/rural background
Educational Status	12.05	3	0.017	Education level showed a significant association with medication status
Duration of Disease	32.31	4	< 0.001	Medication status was significantly associated with duration of illness
Socioeconomic Status	0.045	3	0.997	No significant association was observed between SES and medication status

Table 4 demonstrates the comparison of symptom severity by medication status. Medicated patients had a significantly lower mean BPRS score (50.51 ± 10.58) than unmedicated patients (78.24 ± 10.57). This difference was highly significant (t = -12.90, df = 86.25, p < 0.0001) with a very large effect size (Cohen’s d = -2.60), confirming both statistical and clinical relevance.

**Table 4:** Symptom severity by medication BPRS = Brief Psychiatric Rating Scale

Group	Number	Mean BPRS	SD	Welch’s t-test	DF	P-value
Medicated	59	50.51	10.58	-12.90	86.25	< 0.0001
Unmedicated	41	78.24	10.57			

Table 5 highlights the differences in inflammatory markers by treatment status. The mean NLR was markedly higher in unmedicated patients (8.94 ± 5.08) compared to medicated patients (4.57 ± 1.90). Welch’s t-test confirmed this difference to be highly significant (t = -5.26, df = 48, p < 0.0001), with a large effect size (Cohen’s d = -1.23), indicating that antipsychotic treatment may reduce systemic inflammation.

**Table 5:** Inflammatory markers by medication status NLR = neutrophil–lymphocyte ratio, DF = degree of freedom, SD = standard deviation

Group	Number	Mean NLR	SD	DF	Welch’s t-test	P-value
Medicated	59	4.57	1.9	48	-5.26	< 0.0001
Unmedicated	41	8.94	5.08			

Table 6 summarizes correlations between blood parameters and BPRS scores. Absolute neutrophil count (ANC) correlated strongly and positively with symptom severity (r = +0.81), while absolute monocyte count (AMC) showed a moderate positive correlation (r = +0.63). Absolute lymphocyte count (ALC) had a strong negative correlation (r = -0.85), indicating that reduced lymphocyte levels were associated with greater severity. 

**Table 6:** Correlation of blood parameters with symptom severity

Blood Parameter	Correlation (r)	Interpretation
Absolute Neutrophil Count (ANC)	+0.81	Strong positive correlation
Absolute Lymphocyte Count (ALC)	-0.85	Strong negative correlation
Absolute Monocyte Count (AMC)	+0.63	Moderate positive correlation

**Figure 1:**
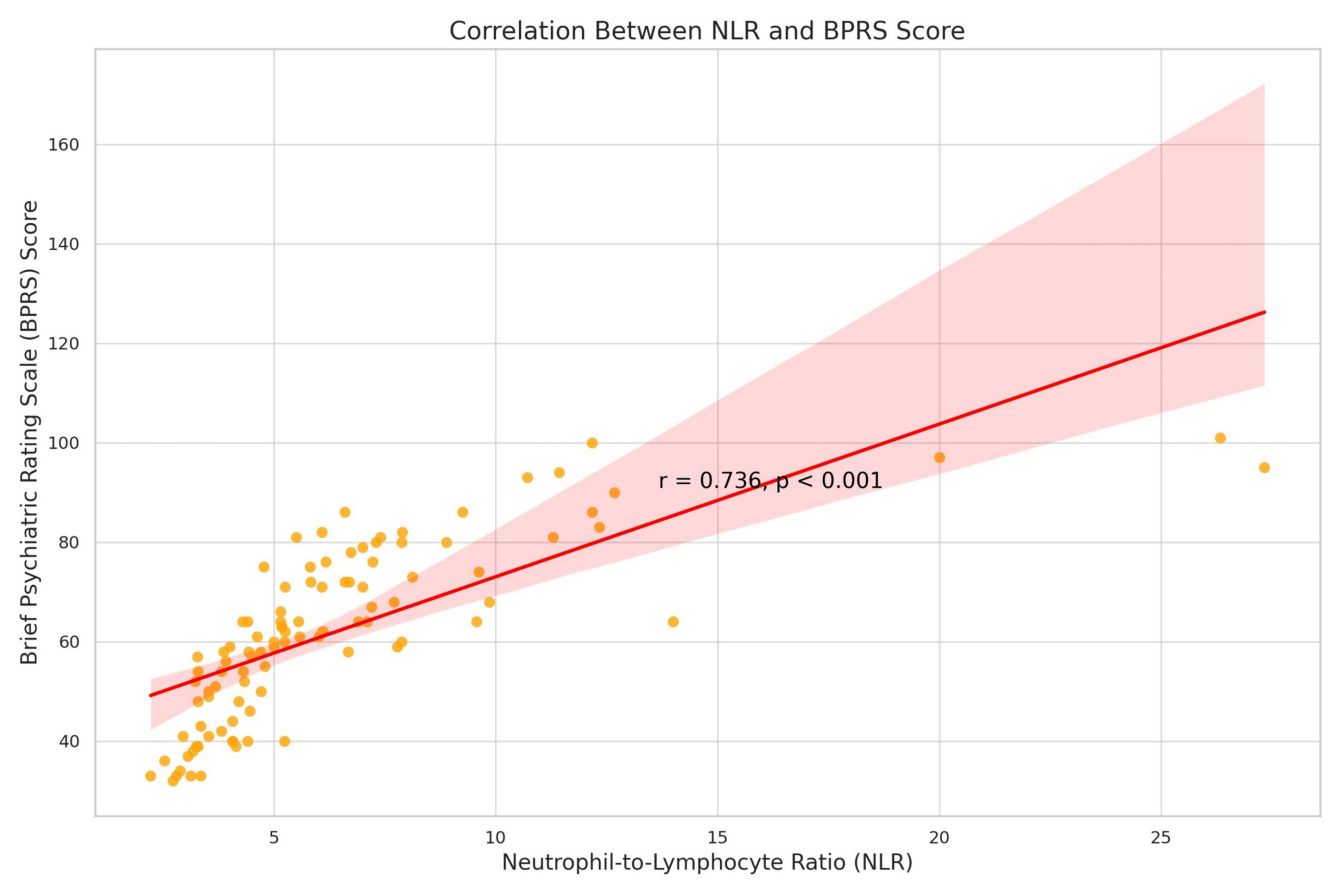
Correlation between NLR and BPRS NLR = neutrophil-lymphocyte ratio, BPRS = Brief Psychiatric Rating Scale, r = Pearson correlation coefficient

Figure 1 illustrates the positive correlation between NLR and BPRS scores. A clear upward trend was observed (r = +0.736, p < 0.001), demonstrating that higher NLR values were strongly associated with more severe psychiatric symptoms (Figure 1).

## Discussion

The present study was a cross-sectional, hospital-based observational study including 100 patients with schizophrenia diagnosed according to ICD-10 criteria, recruited from both inpatient and outpatient settings. The majority of patients were middle-aged adults, with 37% in the 41-50 year range and 34% in the 51-60 year range. This reinforces that schizophrenia remains a major burden during midlife, with significant consequences for occupational and social functioning. Similar age distributions have been reported in prior studies; Mazza et al. (2019) noted an average age range of 30-45 years across psychotic cohorts [27], while Wang et al. (2024) described younger, early-onset first-episode patients with a mean age of 24.1 years [36].

A clear male predominance was also observed, with 73% of participants being male. This finding is consistent with established epidemiological evidence: schizophrenia is diagnosed approximately 1.4 times more often in men than in women, with earlier onset in men (18-25 years) compared to women (25-35 years) (Picchioni and Murray, 2007) [37]. Ochoa et al. (2012) [38] and Giordano et al. (2021) [39] similarly reported that males tend to present earlier, while females often develop symptoms later in their late 20s or early 30s. More recent studies reinforce this male predominance; Mazza et al. (2019), in their meta-analysis of 683 psychotic patients, found a consistent male majority across included studies while Wang et al. (2024) reported 56.7% male patients among first-episode medication-naïve cases [27,36].

In the present study, 59% of patients were receiving antipsychotic medications, whereas 41% remained untreated at the time of evaluation. This distribution enabled meaningful comparison of systemic inflammatory markers between treated and untreated groups, which is clinically important for understanding how antipsychotic therapy may modulate systemic inflammation and disease severity. Beyond treatment status, education and illness duration significantly influenced access to therapy. Patients with higher education were more likely to receive medication, while those with lower education often remained untreated. Furthermore, all newly diagnosed cases were untreated, with treatment uptake increasing progressively with longer illness duration. These disparities mirror challenges in treatment access and adherence documented in Indian settings, where rural and disadvantaged populations face persistent treatment gaps [40-42].

The present study demonstrated a strong positive correlation between NLR and schizophrenia severity as measured by the BPRS (r = 0.736, p < 0.001). Comparable associations have been reported across diverse cohorts. Wang et al. (2024) found that higher NLR values correlated with PANSS total scores in first-episode, medication-naïve patients (r = 0.33, p = 0.001) [36], while Sugita et al. (2024) demonstrated significantly higher NLR in more severely symptomatic or undertreated patients [30]. Similar findings were noted in meta-analyses by Mazza et al. (2020) [27] and Karageorgiou et al. (2019) [28], reinforcing that systemic inflammation, reflected in elevated NLR, is robustly associated with schizophrenia severity. Other independent studies have also supported this relationship: Kulaksizoglu and Kulaksizoglu (2016) showed significant positive correlations between NLR and PANSS scores [43]; Surati et al. (2022) observed elevated NLR paralleling symptom burden in chronic cases [44]; and Zhang et al. (2017) highlighted that higher inflammatory indices, such as increased hs-CRP/IL-10 ratio, were linked to greater aggression and psychosis severity [45]. Cytokine research further provides biological plausibility, with elevated IL-6 and TNF-α consistently associated with the intensity of positive symptoms, including hallucinations and delusions [45]. Collectively, these findings underscore the reliability of NLR as an accessible and inexpensive adjunctive biomarker for monitoring symptom severity in schizophrenia.

The study also had notable strengths. Inclusion of both treated and untreated patients allowed meaningful comparisons and highlighted the modulatory effect of antipsychotic therapy on systemic inflammation. The use of the BPRS, a standardized and validated tool, together with the NLR, a simple biomarker derived from routine blood counts, enhances the clinical applicability of the findings. The sample size was determined using an analysis-appropriate power calculation for continuous outcomes, and the inclusion of 100 participants provided robust power to detect both group differences and correlations. In addition, detailed sociodemographic and clinical profiling, including education and socioeconomic status, offered important contextual insights. Rigorous eligibility criteria and statistical analysis further strengthened the validity of the study and contributed reliable data from an Indian population to the global literature on inflammation in schizophrenia.

At the same time, certain limitations must be acknowledged. The use of convenience sampling may have introduced selection bias. Although patients with major comorbidities and active infections were excluded, unrecognized subclinical inflammatory conditions or lifestyle factors could have influenced blood counts. The cross-sectional design restricted interpretation to associations rather than causation, underscoring the need for prospective and treatment-response studies. Additional immune markers such as cytokines were not assessed, limiting the ability to confirm whether elevated NLR is an independent indicator of immune dysregulation. Furthermore, neutrophil counts are highly variable with a short lifespan, but repeat measurements were not performed. Psychiatric assessment was conducted by a single consultant psychiatrist, reducing inter-rater variability but also precluding evaluation of inter-rater reliability.

Despite these limitations, the findings provide important directions for future research. Longitudinal and multicentric studies, particularly treatment-response investigations, are needed to clarify temporal and causal associations between systemic inflammation and schizophrenia severity. While the present findings highlight the clinical relevance of NLR, the precise mechanisms by which peripheral inflammation relates to brain pathology and symptom expression remain unclear. Studies integrating NLR with other easily measurable peripheral biomarkers (such as C-reactive protein and cytokine profiles) may help explain these pathways. Furthermore, careful documentation of antipsychotic drug type, dose, and duration is required to disentangle treatment-related effects on inflammatory status. Finally, predictive models that combine NLR with socio-demographic and clinical characteristics could advance its application as a low-cost adjunctive tool for risk stratification and personalized management in schizophrenia.

## Conclusions

The findings of this study indicate that elevated NLR is strongly associated with greater schizophrenia severity, with untreated patients showing the highest values. Although causal inference cannot be established due to the cross-sectional design, the results support the potential utility of NLR as an adjunctive biomarker for routine clinical practice. Routine NLR monitoring may complement psychiatric assessment, may help identify patients at risk of severe symptoms, particularly those not receiving treatment, and may guide timely interventions. As NLR is inexpensive and readily available from routine blood counts, it holds promise as a cost-effective tool for risk stratification and personalized management, especially in resource-limited settings. Future prospective and treatment-response studies integrating additional inflammatory biomarkers (e.g., cytokines, CRP) are warranted to validate these observations and clarify underlying mechanisms.
